# Circulating Tumour Cell Biomarkers in Head and Neck Cancer: Current Progress and Future Prospects

**DOI:** 10.3390/cancers11081115

**Published:** 2019-08-05

**Authors:** Karl Payne, Jill Brooks, Rachel Spruce, Nikolaos Batis, Graham Taylor, Paul Nankivell, Hisham Mehanna

**Affiliations:** 1 Institute of Head and Neck Studies and Education, University of Birmingham, Birmingham B15 2TT, UK; 2 Institute of Immunology and Immunotherapy, University of Birmingham, Birmingham B15 2TT, UK

**Keywords:** head and neck cancer, circulating tumour cell, biomarker

## Abstract

Head and neck cancer (HNC) continues to carry a significant burden of disease both for patients and health services. Facilitating biomarker-led treatment decisions is critical to improve outcomes in this group and deliver therapy tailored to the individual tumour biological profile. One solution to develop such biomarkers is a liquid biopsy analysing circulating tumour cells (CTCs)—providing a non-invasive and dynamic assessment of tumour specific alterations in ‘real-time’. A major obstacle to implementing such a test is the standardisation of CTC isolation methods and subsequent down-stream analysis. Several options are available, with a recent shift in vogue from positive-selection marker-dependent isolation systems to marker-independent negative-selection techniques. HNC single-CTC characterisation, including single-cell sequencing, to identify actionable mutations and gene-expression signatures has the potential to both guide the understanding of patient tumour heterogeneity and support the adoption of personalised medicine strategies. Microfluidic approaches for isolating CTCs and cell clusters are emerging as novel technologies which can be incorporated with computational platforms to complement current diagnostic and prognostic strategies. We review the current literature to assess progress regarding CTC biomarkers in HNC and potential avenues for future translational research and clinical implementation.

## 1. Introduction

Despite advances in treatment and patient stratification, head and neck cancer continues to carry a significant burden of disease both for patients and health services. Global incidence is estimated to be 650,000 per annum [1] and this number has risen by over 30% since the early 1990s [2], particularly in younger patients and females, due to human-papillomavirus (HPV) driven disease without the traditional risk factors of tobacco or alcohol exposure. Five-year survival remains static in the region of 50–60% with only a slight increase over the past few decades, especially for non-HPV related cancers, with recurrence or metastasis (R/M) expected in up to 30% of patients [3].

To combat such a burden of disease and achieve improved treatment outcomes, modern oncology is shifting from empirical treatment strategies to biomarker-led treatment models based upon the molecular profile of the tumour. So called ‘personalised medicine’ is the panacea of cancer therapy—choosing targeted therapies based on patient-specific tumour genetic data and biology. However, such a goal first requires a means to assess such tumour characteristics. Not only do we require the sensitivity and specificity of a test to guide treatment, but we must be able to repeat this test serially to monitor tumour progression (including genetic clonal evolution) or treatment response/resistance. 

**Figure 1:**
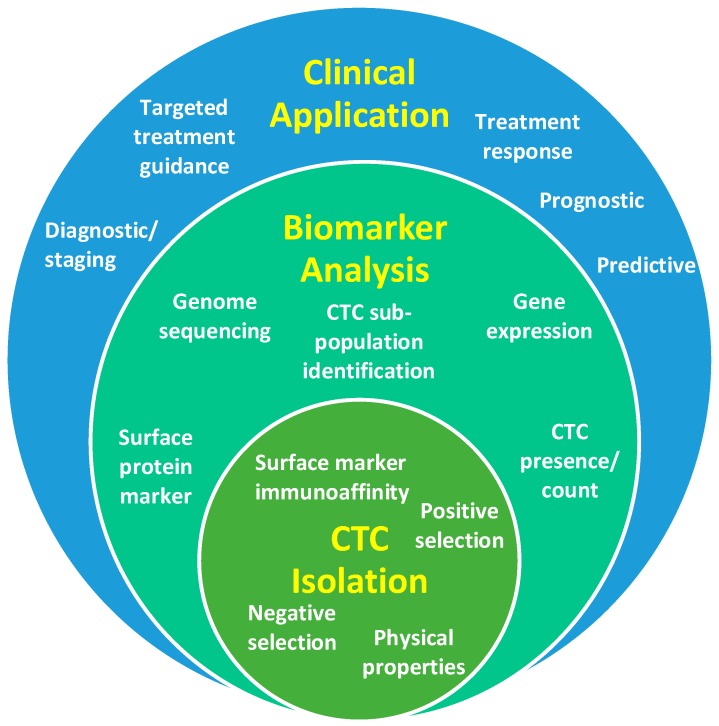
Diagrammatic representation of the relationship between circulating tumour cell (CTC) isolation strategies, biomarker outputs and clinical applications.

A proposed solution to this problem is a ‘liquid biopsy’—using a blood test to detect and analyse circulating tumour cells (CTCs) and/or circulating fragments of tumour DNA (ctDNA) to provide cancer biomarkers. This approach holds the promise to facilitate accurate patient risk stratification, guide treatment selection, predict response, and identify the failure of treatment early, thereby allowing a timely shift of therapeutic strategy [4,5]. In contrast to a tissue biopsy, blood sampling is inexpensive, therefore the main costs of a liquid biopsy are the associated laboratory studies and the downstream data analysis. The ideal liquid biopsy should be inexpensive enough to be repeated at multiple time-points during treatment, to provide a ‘dynamic’ picture of the tumour burden and the evolving mechanism of resistance to therapy [4,6]. Evidence suggests this could significantly improve outcomes in head and neck squamous cell carcinoma (HNSCC) [4], while reducing patient morbidity from unnecessary treatments, and optimising use of healthcare resources [7]. It is essential to the understanding of a CTC liquid biopsy that there is an appreciation of the impact different CTC isolation methods can have on the availability of various biomarkers and how these can translate to clinical practice (Figure 1). Therefore, this review article discusses the biology, isolation, and analysis of a CTC liquid biopsy with a focus on clinical biomarker discovery—it also highlights future prospects in this growing field of research. 

## 2. CTC Biology

CTCs are cells derived from a tumour mass (primary or metastatic) that have entered the vascular circulation, first reported as early as 1869 [8]. The exact mechanism of this process remains unknown, likely a combination of shedding directly into ‘leaky’ vasculature as the tumour grows and induces angiogenesis, indirect lymphatic spread, and the active migration of tumour cells [9,10]. These migratory cells have acquired a ‘plasticity’ via a full or partial epithelial-to-mesenchymal transition (EMT), which results in a loss of inter-cellular adhesion and increased cell motility, allowing them to traverse the basement membrane and undergo intravasation [11]. Research into the impact of a hypoxic tumour microenvironment and the upregulation of key hypoxia-inducible factor genes is continuing to elucidate its role within this metastatic pathway—promoting tumour progression and radiation resistance, with new evidence of the relationship of tumour hypoxia to EMT [12]. Therefore, research into EMT in CTCs is crucial to understand and therapeutically combat cancer cell dissemination and subsequent metastasis formation.

Once within the circulation CTCs must survive difficult conditions, including the shear forces of turbulent blood flow as well as immunological surveillance. For these reasons, the half-life of CTCs is estimated to be low—in the region of several hours [8]. In addition to single cells, CTCs have been identified in clusters, termed ‘circulating tumour micro-emboli’, bound to various stromal and white blood cells (WBCs), e.g., neutrophils [13]. Recent evidence has suggested that these tumour micro-emboli have a higher metastatic potential than single CTCs [14] and that these cell aggregates provide protection from shear forces and immune surveillance [15], also acting as stimulatory CTC escorts driving metastasis [13]. Given the number of CTCs in cancer patients, it is clear that not every CTC forms a metastasis (likely <0.01%), however those cells with mixed phenotypes appear to survive longer in the circulation [16]. Research continues to investigate if specific CTC characteristics are implicated in patterns of metastasis, e.g., the recent demonstration of a brain metastasis expression signature in CTCs of breast cancer patients [17].

## 3. CTC Isolation Methods

A significant challenge in enriching and isolating CTC populations is the rarity of these cells when compared to the millions of circulating blood cells. While quoted figures vary, assuming a CTC yield in the tens to hundreds per millilitre of blood, CTCs comprise <0.004% of all mononucleated cells [8], or approximately one in a billion circulating cells [18]. CTC isolation techniques can be divided into two main strategies: those that use physical properties—such as size/deformability, density, electrical properties—to enrich the blood cell population, and those that use biological properties, isolating cells based on their expression of specific proteins markers [8,9]. Antibodies specific for relevant markers of interest are generally used in the latter approaches. Enrichment and isolation by physical or biological means may operate by ‘positive selection’ or ‘marker dependent’ methods—identifying CTCs based on specific characteristics, or by ‘negative selection’ or ‘marker independent’ methods—depleting other cell populations to leave a pure CTC population. 

Some of the first research into CTCs utilised reverse-transcription polymerase-chain reaction (RT-PCR) to identify tumour specific RNA that would demonstrate the presence of CTCs in a blood sample [18]. However, while this technique confirms the presence of CTCs and individual gene expression, it does not allow for the characterisation of individual cells or accurate enumeration. Currently, the only FDA approved device for CTC isolation remains the CellSearch^®^ system, which targets the cell surface antigen—epithelial cell adhesion molecule (EpCAM), enriching via immunomagnetic positive selection. As aforementioned, a crucial step in the metastatic pathway is the EMT that creates phenotypically diverse CTCs. Positive selection methods reliant upon targeting epithelial antigens only are therefore inherently biased against detecting ‘non-epithelial’ CTCs, which may be mesenchymal, stem-cell, or a mixture of the above—thus demonstrating a high epithelial CTC specificity but low sensitivity of this technique to detect CTC sub-populations in HNSCC [18]. Alternative methods for multiparameter positive selection include using flow cytometry or fluorescence-activated cell sorting techniques (FACS) [19]. In this technique the blood sample must first undergo a negative selection enrichment step (to remove red blood cells and a large fraction of WBCs) and then immunofluorescent antibodies are chosen to target multiple surface proteins, minimising the bias from ‘epithelial only’ techniques. The downside of using FACS is the potential for cell loss, which, given the low numbers of CTCs expected in a blood sample, may have a significant impact upon the accuracy to use CTC count as a surrogate biomarker. 

**Figure 2:**
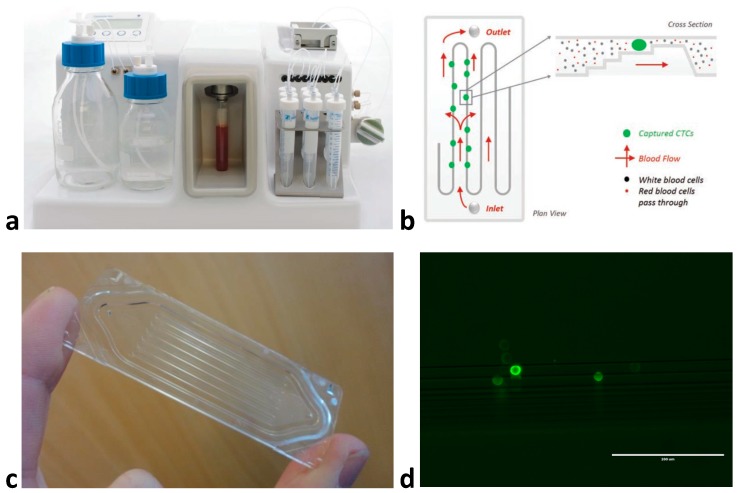
An example of CTC enrichment using a microfluidic device: (**a**) the Parsortix™ (Angle Plc) system, (**b**) diagram of microfluidic flow and cell sorting within the isolation cassette, (**c**) Close-up of the isolation cassette demonstrating tiered multi-channel design, (**d**) head and neck squamous cell carcinoma cells (FaDu cell line) isolated from spiked blood sample using Parsortix™ and stained with pan-cytokeratin antibody (Parsortix™ enriches CTCs ≈ 1000-fold, however anti-CD45 staining is also required to negatively select the contaminating white blood cell population or deplete CD45+ve cells to provide a pure CTC population) at 20× magnification.

Techniques that use physical properties for CTC isolation have largely focused upon microfluidic based approaches to enrich blood samples [8]. The principle behind these techniques is that CTCs are assumed to be larger than the majority of circulating cells. A specially designed microfluidic device/cassette is used to separate cells (Figure 2a–c), with techniques including size-based filtration, size-based streamline sorting—whereby pressurised fluid forces cells into size based ‘streams’—and dielectrophoresis, which uses electrical fields to polarize cells of varying sizes, or combinations of the above [20]. Once enriched, cells must undergo an antibody staining protocol to correctly identify CTCs (Figure 2d). The accepted definition of a CTC in HNSCC is a cell staining positive for an epithelial marker and DAPI (nuclear dye) and negative for CD45 (a leukocyte marker) [10,18]. While EpCAM has been used as an epithelial marker [19,21], cytokeratin (CK) 8, 18, 19 or 20 also appear to be reliable in HNSCC [22,23,24]. In addition to the above protocol, markers for mesenchymal (N-cadherin or Vimentin) and/or stem cell (CD133) phenotypes have been used and are discussed below [24,25]. Often the limiting factor for CTC characterisation is the number of immunofluorescent channels available when using microscopy (usually a maximum of four), hence recent interest in multiparameter techniques such as flow or mass cytometry. 

A recent systematic review revealed 22 studies investigating CTCs in HNSCC [5]. The majority of these studies (15/22) used an immunomagnetic positive selection technique to identify CTCs; within this group the CellSearch^®^ system was most frequently utilized [5]. It can be seen from the above list of complex isolation methods that a direct comparison of data from studies using differing techniques can be difficult. In addition, the protocol for sampling blood is not standardised and the time that can be allowed from collection to processing is unknown (quoted in the region of 3–5 days) as is the impact this may have upon CTC viability. As will be discussed, the number of isolated CTCs varies greatly between positive and negative selection protocols, as does the data output from different CTC characterisation techniques. 

## 4. CTC Count as a Diagnostic and Prognostic Biomarker in HNSCC 

### 4.1. Diagnostic Biomarker of Stage of Disease

A recent systematic review concluded that evidence is equivocal as to whether CTCs can diagnostically predict tumour-node-metastasis (TNM) stage in HNSCC [5]. It would seem logical that a larger tumour with a greater T stage, or with nodal or distant metastases representing increased burden of disease would release more CTCs into the bloodstream. However, this may not be the case if other factors, such as patterns of lymphovascular infiltration, tumour differentiation or anatomical site also govern CTC shedding. Hristozova et al. evaluated CTC count in 42 patients with locally advanced inoperable HNSCC, using flow cytometry [26]. While patients with a greater T status had increased numbers of CTCs/mL this was not statistically significant. They did however demonstrate a significant correlation (*p* = 0.007) between nodal stage and CTC count, when defining two groups (N0-N2a and N2b-N3). The presence of CTCs was greatly increased in oral cavity and oropharyngeal cancers compared to other anatomical sites but had no association with HPV status. It should be noted that only 43% of patients (18/42) had detectable CTCs, with a mean CTC count of 1.7. It is unclear if this is a true reflection of CTC activity in relation to tumour burden, or the sensitivity of the flow cytometry detection method to detect low cell numbers. In a similar study, Kawada et al. used a micro-filter (CellSieve) to detect CTCs in 32 patients, achieving a higher detection rate of 90.6% (29/32) [23]. They demonstrated that advanced stage of disease (III–IV) and T status (T3–4) was significantly associated with increased CTC count (*p* < 0.05 and *p* < 0.01). Contrary to Hristozova, they found no relationship with nodal status. Both Hristozova and Kawada were able to show a significant decrease in CTC count immediately post-treatment, demonstrating the potential utility of this assay in post-treatment monitoring. 

### 4.2. Prognostic Biomarker of Survival Outcomes and Treatment Response 

Several studies have investigated survival outcomes and the use of CTC presence/count as a prognostic marker. Jatana et al. measured CTC count at the time of surgery in 48 patients (the majority of which, 65%, were late stage III/IV disease) using a negative depletion method, with a mean follow-up of 19 months [27]. They reported that the presence of CTCs (detectable in 71% of patients) was significantly correlated with decreased disease-free survival (DFS), and those with >25 CTCs/mL had a worse clinical outcome. Using the CellSearch^®^ system in a R/M 53 patient cohort, Grisanti et al. demonstrated that a baseline CTC count >2 was a prognostic indicator of worse progression-free (PFS) and overall survival (OS) (*p* < 0.0005) [28]. However, these results are tempered by only 26% of patients having detectable CTCs at baseline and low CTC counts (mean 1, range 0–43). In the largest study cohort to-date, Tinhofer et al. used RT-PCR of epidermal growth factor receptor (EGFR) transcripts to analyse the presence of CTCs in 144 locally advanced HNC patients who had undergone previous primary surgical resection [29]. They detected evidence of CTCs in 29% (42/144) of patients, but with a median follow-up of 34 months were unable to correlate CTC positivity with DFS or OS, independent of the modality of adjuvant treatment. However, when they separated patients into oropharyngeal carcinoma (OPC) and non-OPC groups, CTC presence was a prognostic marker of worse DFS and OS in non-OPC patients, but conversely was a marker of improved DFS and OS in OPC patients. While an obvious reason for this difference would be the confounding factor of improved outcomes in HPV-driven OPC, they were unable to correlate CTC positivity with p16 status. Such mixed results are likely a product of heterogenous EGFR expression in HNSCC and emphasise a need for negative selection protocols. 

Despite their seemingly pivotal role in metastasis, few studies have investigated CTC clusters and survival outcomes. Kulasinghe et al. recently demonstrated that in a cohort of 60 HNC patients, 25% exhibited CTC clusters (ranging from 1–3 clusters/5 mL—consisting of 3–13 cells) [15]. Of note, all patients with CTC clusters had advanced stage IV disease and there was no correlation between single-CTC and cluster presence (10/20 patients single-CTC +ve were cluster -ve and 5/15 patients cluster +ve were single-CTC -ve). However, the presence of CTC clusters was significantly associated with distant metastatic disease (*p* = 0.0313) [15]. Such data provides early evidence that this entity could be a meaningful prognostic marker and should also be a focus of future research. 

The translational clinical benefit of a CTC liquid biopsy over current practice is the ability to perform serial measurements to monitor and/or guide treatment. As a reference, CTC count in HNSCC appears broadly similar to other cancers [9], for example cut-off values of ≥5 or ≥3 CTCs (per 7.5 mL blood) demonstrated prognostic significance in breast cancer [30] and colorectal cancer [31] respectively. One of the first groups to consider how CTCs could be used as predictive markers of treatment response were Buglione et al. [22]. They evaluated CTCs at multiple treatment time-points (before, during and after treatment) in 73 HNC patients using CellSearch^®^. Unfortunately, their conclusions were limited due to only 15% (11/73) of patients having detectable CTCs present at diagnosis. Despite this, they demonstrated that when CTCs were absent at diagnosis or decreased below limits of detection in post-treatment samples the patients were significantly more likely (*p* = 0.017) to have a complete/partial treatment response, compared to those who had an increase in CTC count and did not respond to treatment. Inhestern et al. used flow cytometry to assess CTC count before, during, and after induction chemotherapy, surgery, and adjuvant chemoradiotherapy (CRT) in 40 HNSCC patients [21]. Eighty percent (32/40) of patients were positive for CTCs during treatment. Their approach was to calculate median CTC count values across all patients at each time-point (baseline, pre- and post-treatment) as a reference point for comparison. Patients with a baseline CTC count > median had significantly worse recurrence-free survival (RFS) (*p* = 0.025), and a maximal CTC count > median at any point during treatment was significantly associated with worse OS (*p* = 0.049). In a similar study, Wang et al. investigated CTC count (using a negative depletion and flow cytometry detection technique) immediately before and 2–4 weeks after CRT treatment in 47 HNSCC patients. Decline in CTC count post-treatment was significantly associated with PFS and OS (*p* = 0.01 and 0.013) [19]. Evidence from these types of studies starts to illustrate how serial measurements of CTC count could have prognostic and/or predictive value during treatment. 

## 5. CTC Characterisation in HNSCC

While the primary goal of early research was to identify and count CTCs in HNSCC, current research is focused on providing intact single cells that can be further characterised to provide protein-expression and genetic level data. One reason for static survival rates in HNSCC is the considerable genetic heterogeneity of this disease [32], which in turn correlates with decreased OS [33]. Distinct regions of a tumour may contain different genetic mutations driving growth, potential therapy resistance and recurrence [33]. The current method of a random diagnostic tissue biopsy may exhibit sampling bias due to ‘spatial heterogeneity’ and fail to represent the entire mutational landscape and is therefore inadequate [34]. Moreover, a tissue biopsy may be difficult or impossible to obtain, for example in R/M patients, or be too morbid for a patient to undergo on a serial basis, leaving the clinician blind as to the emergence of treatment resistance [35]. 

Developing improved negative selection CTC isolation methods has allowed researchers to investigate multiple CTC surface protein markers in HNSCC, beyond the conventional epithelial markers used in positive selection techniques. This has provided evidence that distinct phenotypic CTC sub-populations exist, that may be of importance with regard to the previously discussed EMT and tumour metastatic potential, in addition to guiding targeted therapy. Balasubramanian et al. investigated expression of multiple surface markers in a small patient cohort, including—CK 8,18,19 and EpCAM (epithelial), N-cadherin and Vimentin (mesenchymal), EGFR and CD44 (cell migration marker) [25]. They demonstrated multiple phenotypic patterns within this marker panel, for the first time highlighting that over a third of CTCs in HNSCC were negative for epithelial markers but positive for mesenchymal markers. These cell populations would have been missed with previous epithelial-based positive selection techniques. Weller et al. corroborated these findings that around a third of CTCs in HNSCC had a mesenchymal phenotype [24]. They investigated epithelial, mesenchymal and cancer stem cell markers in CTCs isolated from 10 HNSCC patients, with long term follow-up data. They found that CTCs were present that exhibited both epithelial (CK) and mesenchymal (N-cadherin) markers, in addition to combinations of epithelial or mesenchymal CTCs that were also positive for stem cell marker CD133. This mixed marker expression is likely due to the epithelial-mesenchymal change being a transitional process, as opposed to a ‘switch’. Analysis of blood samples taken before and after surgery showed the expected decrease in CTC numbers post-treatment; interestingly, the presence of mesenchymal CTCs post-resection was associated with significantly decreased OS (474 vs. 235 days, *p* = 0.04). 

Podoplanin (PDPN) is involved in lymphangiogenesis and is seen as a marker of poor prognosis and a potential therapeutic target in several cancers. Hsieh et al. investigated CTC expression of PDPN in 53 locally advanced/metastatic HNSCC patients [36]. Their technique was to positively select epithelial (EpCAM) cells and then test for PDPN expression. With a median follow-up of 10.5 months, they found no significant relationship between PFS or OS and the total number of PDPN positive CTCs. However, if they calculated the proportion of PDPN positive/epithelial positive CTCs, they found that patients with >20% PDPN positive CTCs had significantly worse PFS and OS (*p* = 0.006 and *p* = 0.008). Clearly this demonstrated that if tumours displayed this marker of invasive potential the clinical outcome was worse, possibly due to the increased metastatic potential of these CTC sub-populations. 

## 6. Biomarker Led Stratification and Targeted Treatment Guidance 

Recently, targeted immunotherapies have produced dramatic improvements of outcomes in a variety of tumour types, and show significant promise in HNSCC [37]. However, targeted-immunotherapy treatments are not without potential toxicity and identifying patients who will not respond would reduce morbidity from ineffective treatments and reduce the significant cost burden of these expensive treatments. Across all cancers evidence demonstrates that biomarker-led selection of therapies results in better responses to treatment [38], unfortunately, HNSCC currently has no marker that can predict response to and guide treatment. Therefore, novel biomarkers are required—the characterisation of CTCs from a liquid biopsy holds particular promise in this regard [10,18]. A recent study in prostate cancer correlated low CTC phenotypic heterogeneity with improved OS in a targeted therapy group (androgen receptor inhibitor) and conversely high CTC phenotypic heterogeneity was associated with improved OS in a chemotherapy group [39]. 

R/M HNSCC patients have a dismal prognosis, often confined to palliative treatment [3], therefore any test to better guide treatment in this group could have a profound impact upon patient care. Recent evidence has demonstrated programmed death-ligand 1 (PD-L1) expression as a potential biomarker for immunotherapy in HNSCC—with the combined positive score (evaluating tumour *and* tumour-associated immune cell PD-L1 expression) an accepted method of patient stratification for anti-PD1 immunotherapy in R/M patients [40]. However, of note is previous evidence describing anti-PD1 treatment response in patients with PD-L1 negative tumour biopsies (<1% expression) [41]. Chikamatsu et al. used RT-PCR to detect CTC expression (via RNA transcript detection) of eight target genes (*CK19, EpCAM, EGFR, c-Met, PD-L1, PD-L2* and *CD47*) in blood samples from 30 R/M patients, of which 24 patients were CTC-positive [42]. The key finding from this study was heterogeneity of PD-L1 expression between tumour samples (from solid tissue biopsies) and CTCs—in 10 patients the tumour was negative for PD-L1 and the CTC sample demonstrated PD-L1 expression. For the first time, this study demonstrates the translational clinical utility of a CTC liquid biopsy to identify HNSCC tumour heterogeneity not seen on a tissue biopsy, that could guide immunotherapy in R/M patients. Also investigating immune checkpoint markers by utilizing mRNA RT-qPCR, Strati et al. demonstrated PD-L1 overexpression in EpCAM +ve CTCs in 94 locally advanced HNSCC patients [43]. Twenty-four patients (25.5%) exhibited CTC PD-L1 overexpression at baseline. Available data of CTC PD-L1 overexpression from 36 patients demonstrated that 8 of 13 patients remained PD-L1+ve after completion of treatment and two patients who were PD-L1-ve at baseline developed PD-L1 overexpression during treatment. CTC PD-L1-ve status at both baseline (*p* = 0.085) and the end of treatment (*p* = 0.001) was associated with a complete response to treatment. Furthermore, in longitudinal data from 54 patients, CTC PD-L1 overexpression post-treatment was an independent prognostic factor for PFS (*p* = 0.001) and OS (*p* < 0.001). While much of the discussion to-date has been in R/M patient groups—the conclusion these authors made was that CTC PD-L1 status could be used as a prognostic biomarker to guide primary adjuvant-immunotherapy in HNSCC patients. 

It is interesting to compare the above evidence of PD-L1 heterogeneity between positive CTCs and negative tumour samples in HNSCC and findings from trials of targeted treatment in other cancer types. For example, a recent randomised phase II trial of Trastuzumab (human epidermal growth factor receptor 2 (HER2) inhibitor) efficacy in HER2-ve breast cancer reported that 89% (51/57) of patients demonstrated HER2+ve CTCs, despite confirmed tumour HER2-ve status [44]. The clinical significance of HER2 as a therapeutic target demonstrating heterogeneity between CTCs and tumour samples was unclear, despite improved DFS in the treatment arm. Cancer cells with stem cell-like properties are a rare sub-population that may play a key role in both spatial and temporal heterogeneity—being responsible for tumour initiation, maintenance and contributing to treatment resistance and metastatic spread. The discovery of CTCs with stem cell characteristics in HNSCC, in addition to CTCs with mixed stem cell and epithelial/mesenchymal phenotypes [24] demonstrates this potential pathway. However, evidence of such ‘inter-compartmental’ heterogeneity between tumour and CTCs poses several questions—Is this a true representation of tumour clonal evolution causing disease progression, or merely a demonstration of the inherent sampling bias in a tissue biopsy to detect intra-tumoural heterogeneity? It remains to be seen if CTC derived biomarkers will provide more accurate prognostic and predictive risk stratification than tissue derived markers, but most likely an amalgamation of compartmental biomarkers, akin to the PD-L1 combined prognostic score, will be required. 

## 7. Single-Cell Sequencing—The Future of CTC Analysis

### 7.1. Approaches to CTC Genetic Sequencing 

The ability to derive ‘multi-omic’—that is, proteomic, genomic, epigenomic and transcriptomic—level data from cancer cells is a crucial step in identifying treatment-induced clonal selection and resistance in HNSCC [45]. A CTC liquid biopsy is a potential avenue to obtain ‘real-time’ tumour-specific multi-omic data that could allow the therapeutic targeting of genetic subclones with potential phenotypic advantage(s) leading to treatment resistance [16]. One option to derive genomic/transcriptomic data from CTCs is to ‘pool’ isolated cells, extract DNA/RNA and then bulk sequence—as has been demonstrated in lung cancer to detect serial changes in EGFR mutations during treatment [46]. However, to detect multi-omic heterogeneity within CTC sub-populations requires the clarity of sequencing at the single-cell level, with the ensuing complications associated with the accurate isolation and single-cell sorting of CTCs. The benefit of single-cell sequencing to identify intra-tumoural genomic heterogeneity in HNSCC has been reported, along with its potential to impact therapeutic decision making [47]. However, to-date no published evidence exists for similar progress of single-CTC sequencing in HNSCC, although groups are working to this aim. Progress in other cancer types is outlined below. For the purposes of discussion, the output from single-CTC sequencing research can be simplified as either that producing information regarding an actionable mutation for potential treatment guidance, or that which creates a profile or signature related to a known cancer pathway for clinical risk prognostication i.e. metastasis association. 

### 7.2. Single-CTC Genome Sequencing 

Several cancers, including lung, breast, colorectal and prostate, among others, have achieved CTC genetic sequencing at a single-cell level [16]. Heitzer et al. sequenced 37 CTCs from six patients with colorectal cancer, identifying copy-number alterations present in both tumour and CTC samples [48]. Samples from two patients underwent single-CTC sequencing using a targeted 68-gene panel, also sequencing primary tumour and metastasis samples. Notably, 20 mutations were found exclusively within the CTCs. However, on further in-depth sequencing of tissue samples they were able to identify the majority (17/20) of these CTC mutations as sub-clones within the cancer. In a similar study, Shaw et al. sequenced 40 CTCs from five metastatic breast cancer patients [49]. Genomic heterogeneity was evident within the CTC population for *PIK3CA, TP53, ESR1* and *KRAS* mutations. In addition, CTCs demonstrated ESR1 and KRAS mutations not found in matched tumour samples. The authors postulated such tumour-CTC heterogeneity may represent disease progression related sub-clonal evolution, and potential actionable mutations not seen on tissue biopsies. 

### 7.3. Single-CTC Differential Gene-Expression

Despite the aforementioned identification of tumour genomic heterogeneity in CTCs, the true advantage of single-CTC sequencing potentially lies in the identification of gene-expression variation. Transcriptomic analysis of CTCs allows the elucidation of signaling pathways responsible for protein-expression variance governing CTC sub-populations; in turn, providing a picture of disseminated cancer cell biology and potential therapeutic targets [16,50]. Several groups have investigated EMT-related gene expression in CTCs. In a study of eight patients with prostate cancer, Chen et al. used RT-PCR to investigate expression of 84 EMT-related genes in 38 single-CTCs [51]. They were able to show increased CTC metastatic potential through upregulation of several key EMT-related genes, with a heterogeneous expression pattern between CTC populations. Of clinical note from this study was a defined subset of 18 EMT-related genes (e.g., *PTPRN2*, *ALDH1*, *ESR2*, and *WNT5A)* that separated castration-resistant patients from castration-sensitive patients (*p* < 0.001), demonstrating a potential prognostic biomarker signature. Powell et al. profiled the expression of 87 genes within 105 single-CTCs from 35 breast cancer patients (14 primary and 21 metastatic) [52]. For the first time this group were able to define signatures of gene-expression within CTCs that correlated to EMT and clinical metastasis. These findings were echoed by Gorges et al. who performed single-cell RNA qPCR in a cohort of 55 CTCs from five breast and prostate cancer patients. In each patient CTCs were able to be grouped into at least two sub-population clusters based on differential gene-expression related to multiple cancer pathways—including EMT, DNA repair, treatment resistance, stemness and tumour progression [50]. These authors suggest that the identification of such metastatic cell diversity could effectively guide treatment selection, particularly in advanced stage and R/M cancer patients.

A novel approach has recently been demonstrated by Gkountela et al. who investigated CTC methylation profiles in 43 breast cancer patients [53]. For the first time, direct comparison of single-CTCs and CTC clusters demonstrated heterogeneous patterns of hyper- and hypomethylation correlated to clinical outcome. A group of stemness and proliferation associated transcription factor binding sites (*OCT4, NANOG, SOX2, and SIN3A*) were hypomethylated in cell clusters, but conversely hypermethylated in single-CTCs. The hypomethylated CTC-cluster signature correlated to decreased PFS (*p* < 0.05), but no association between single-CTC methylation and PFS was evident. In what should be regarded as a landmark paper, Szczerba et al. were able to isolate CTC clusters from breast cancer patients and perform single-cell RNA sequencing on both CTCs and associated WBCs from these clusters [13]. Of those WBCs in CTC clusters, 85.5–91.7% were neutrophils. Single-cell sequencing revealed differential expression of 51 genes, when comparing expression profiles of single circulating CTCs to neutrophil bound CTCs. Neutrophil bound CTCs exhibited upregulation of genes involved in cell cycle and DNA replication pathways in addition to increased cytokine stimulation, conferring increased proliferation and metastatic potential to these cell clusters. The authors described how these cluster bound neutrophils act as ‘escorts’ to CTCs and this CTC-neutrophil interaction is a potential therapeutic target in cancer metastasis. Given the evidence of increased metastatic potential of CTC clusters in HNSCC [15], research into mechanisms of gene regulation in CTC clusters is clearly of prime clinical importance.

### 7.4. Unanswered Questions

Aside from the technical considerations of single-CTC isolation and sequencing protocols, several key questions remain. While from a basic science point of view identifying single-CTC multi-omic heterogeneity is informative to elucidate metastatic pathways, as clinicians we need to know how this data will change the management of our patients. Arguably the greatest question facing the successful translation of single-CTC characterisation into clinical care is what emphasis we put on CTC heterogeneity. As Brouwer et al. eloquently state: “A key issue remains to what extent heterogeneity in the circulating compartment affects therapy outcome and whether one should take a minor subclone into account if it comes to treatment selection”. To answer this question requires a combination of advanced bioinformatic algorithms correlated with further clinical investigation. Put into context, at an acceptable statistical power, detecting a subclone at 10% frequency requires sequencing of approximately 20 CTCs [54]. Furthermore, given the phenotypic heterogeneity within the CTC compartment and the apparent variance in expression between tumour and CTCs—does this heterogeneity reflect adaptation within a hostile circulating environment, as seen with neutrophil-CTC clusters, or is it a true reflection of the tumour mass? It remains to be seen if the rapid advances in CTC isolation and single-cell sequencing technologies will provide answers to these questions in HNC. However, in modern healthcare, the cost implications of such advanced sequencing techniques are a serious consideration and potential barrier to success. 

## 8. Conclusions

This review has provided a discussion of CTC biology and isolation techniques, in particular negative selection microfluidic approaches, while discussing various CTC biomarkers currently available and under investigation in HNSCC. Highlighting how CTC count/presence may serve as a diagnostic marker of tumour, nodal, and overall stage of disease and a prognostic marker of DFS/PFS and OS. Furthermore, phenotypic sub-populations appear to exist within the CTC compartment in HNC and the expression of mesenchymal or mixed phenotypes correlates with worse clinical outcomes. Finally, the utility for CTCs to provide biomarker-led treatment guidance has been discussed, including PD-L1 and targeted-immunotherapy. Undoubtedly, goals of future research will focus upon achieving single-CTC and CTC cluster multi-omic characterisation in HNC, in line with progress in other cancer types. The first step in the clinical translation of this assay is to define genetic variations, gene expression and protein biomarker signatures that underpin CTC sub-populations and their relationship to the primary tumour mass. Subsequently, large scale clinical trials are required to validate these CTC multi-omic biomarkers, correlating to clinical outcomes, and ultimately to guide anti-cancer therapies [55].
